# Automated deep learning segmentation and planning for left-sided breast radiotherapy with minimised adaptations based on dose, TCP and NTCP criteria

**DOI:** 10.1016/j.phro.2026.100961

**Published:** 2026-04-04

**Authors:** Niels van Acht, Andreea Ciobotaru, Maurice van der Sangen, Jacqueline Theuws, Johanna Bluemink, Coen Hurkmans

**Affiliations:** a Department of Radiation Oncology, Catharina Hospital, Eindhoven, the Netherlands; b Department of Electrical Engineering, Eindhoven University of Technology, Eindhoven, the Netherlands; c Department of Biomedical Engineering, Eindhoven University of Technology, Eindhoven, the Netherlands; d Department of Applied Physics and Science Education, Eindhoven University of Technology, Eindhoven, the Netherlands

**Keywords:** Deep learning segmentation, Deep learning planning, Radiotherapy planning, Dosimetry, Tumour control probability, Normal tissue complication probability

## Abstract

**Background and Purpose:**

Deep learning segmentation (DLS) and planning (DLP) models in radiotherapy treatment planning increase consistency and reduce planning time. However, many DL segmentations and plans are manually adjusted. The clinical relevance of these adjustments remains underexplored. This study assesses the clinical relevance of adjustments made to DLS and DLP and proposes a workflow that could optimise treatment planning time while remaining clinically similar.

**Materials and Methods:**

A retrospective analysis of 101 clinically approved structures and treatment plans (CS-CP) for left-sided node-negative breast cancer was conducted, comparing them with three alternative plans varying in DL influence: unadjusted DLS and DLP (DLS-DLP), clinically approved structures with DLP (CS-DLP), and clinically approved targets with DLS organs-at-risk, referred to as proposed structures, and DLP (PS-DLP). Plans were evaluated based on clinical goals, tumour control probability, and normal tissue complication probability. A new workflow, with four planning routes, was designed to assess time savings.

**Results:**

88 % of CS-CP plans met all clinical goals, compared to 43 % (DLS-DLP), 59 % (CS-DLP) and 60 % (PS-DLP). Statistically significant differences were found for most evaluation criteria, with only the D_98_ and tumour control probability showing clinically relevant differences. The new workflow reduced active planning time by 45 min and overall workflow time by 12 h.

**Conclusion:**

The clinical relevance of DLS and DLP adjustments was assessed, revealing that plans generated by a fully DL workflow (DLS-DLP) were clinically acceptable for 43 % of patients. The proposed workflow optimised planning time, enhancing efficiency.

## Highlights

• Four scenarios utilizing varying deep learning segmentations and planning adaptations were compared.
• Setups were evaluated on DVH goals and radiobiological metrics.
• 43 % of plans generated in a fully deep learning workflow met all clinical goals.
• Edits to target delineation influenced the plan, to organs-at-risk not at all.
• New workflow reduces active time (50 %) and in between steps (30 %) on a plan.

## Graphical abstract

**Figure:**
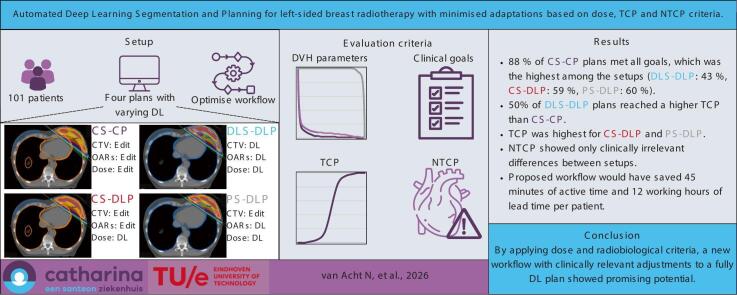

## 1 Introduction

Over the past few years, artificial intelligence (AI) has made its way into the radiotherapy treatment planning workflow [1]. Deep learning (DL) segmentation models have been shown to provide clinically useful segmentations for organs-at-risk (OARs) and for some target volumes. This results in a more consistent, efficient and faster workflow as radiation oncologists (ROs) and radiotherapy technicians (RTTs) no longer need to start from scratch [2], [3], [4], [5].Where DL segmentation models are widely used in clinical practice, DL planning models lag behind in clinical implementation. Conventional treatment planning is labour intensive and associated with large user variability [6], [7], [8]. Some DL planning models have shown to generate clinically acceptable treatment plans with low error rates [9], [10], [11] and generalisability across institutions [12].

For left-sided node-negative breast cancer patients it is possible to utilise both a DL segmentation and DL planning model since the clinical introduction by Bakx et al. [9], [13] in 2021. This workflow typically makes use of several manual checks and gives possibilities for alterations to both the DL segmentation and DL planning model output.

Although Bakx et al. [13] and Almberg et al. [4] reported high clinical acceptability, the degree of adaptations to DL segmentation outputs is still considerable, as shown by Van Acht et al. [14]. Similarly, Besouw et al. [12] showed a statistically significant difference in dose-volume histogram (DVH) parameters between DL plans and clinically accepted plans across institutes. However, what adaptations to the DL segmentation or DL plan are clinically relevant is rarely thoroughly investigated. Irrelevant adaptations increase lead time, as RTTs, ROs and medical physicists (MPs) often need to perform actions in series, waiting on the person prior in the workflow chain. As mentioned by De Kerf et al. [15], to properly evaluate the relevancy of adaptations dose-volume metrics need to be evaluated of plans created directly on the DL segmentation output and compared to clinically approved plans.

Therefore, the goal of this study was to quantify the influence of adaptations of the DL segmentation and DL plan on the final clinically approved breast cancer treatment plan and define a clinical workflow with minimal checks and adaptations while still maintaining clinical acceptability, similar to plans used for treatment.

## 2 Materials and methods

### 2.1 Data

Clinically approved Structures (CS) and Clinically approved Plans (CP) were retrospectively retrieved from 101 fully anonymised patients and used as the gold standard (Table 1). Ethical approval was not required, as the data were retrospective and anonymised. The first patient was treated in November 2020, and the last in July 2025. All treatment plans were created with a commercially available DL segmentation and planning model in RayStation from RaySearch Laboratories AB (dependent on treatment planning date: version 9B, 10B, 12A or 24B). For this treatment protocol, the entire ipsilateral breast was the primary clinical target volume (CTVp) and the lungs, heart and contralateral breast were the OARs. The CTVp was expanded by 5 mm in all directions, with a minimum distance of 5 mm away from the skin, to create the planning target volume (PTVp-Skin05). The target and OARs were the input for the DL planning model.

**Table 1:** Information about the regions-of-interests used in this study. The median (25th percentile (Q1) − 75th percentile (Q3)) was given for the volumes of the CS and DLS as well as the volumetric Dice similarity coefficient (VDSC), surface Dice similarity coefficient with 3 mm tolerance (SDSC 3 mm) and 95th percentile Hausdorff distance (HD95). For the contralateral breast (Breast CL) only volume_DLS_ was available for all 101 patients. The other data was from 12 patients, pointed out by an asterisk in the table.

ROI	volume_CS_ (cm^3^)	volume_DLS_ (cm^3^)	VDSC (−)	SDSC 3 mm (−)	HD95 (mm)
PTVp-Skin05	859 (664–––1126)	885 (660–––1191)	0.95 (0.94–––0.96)	0.9 (0.85–––0.93)	6.0 (4.6–––9.4)
Lungs	4261 (3746–––5056)	4310 (3778–––5092)	0.98 (0.98–––0.99)	0.99 (0.98–––1.0)	1.2(0.0–––1.7)
Heart	652 (560–––763)	625 (544–––702)	0.95 (0.93–––0.96)	0.9 (0.86–––0.92)	9.0 (6.0–––12.0)
Breast CL	1135 (572–––1661)*	684 (493–––998)	1.0 (0.99 – 1.0)*	1.0 (0.99––1.0)*	0.0 (0.0––1.2)*

For the same patients, new DL plans were created for evaluation. All treatment plans were created in line with the intended use of the DL models, for which more information is provided in the Supplementary Materials. The DL planning model consists of a voxel-wise dose prediction model trained on similar clinical goals as used in the institute, followed by dose mimicking optimisation to get a deliverable dose distribution.

### 2.2 Radiotherapy treatment planning workflow

**Fig. 1:**
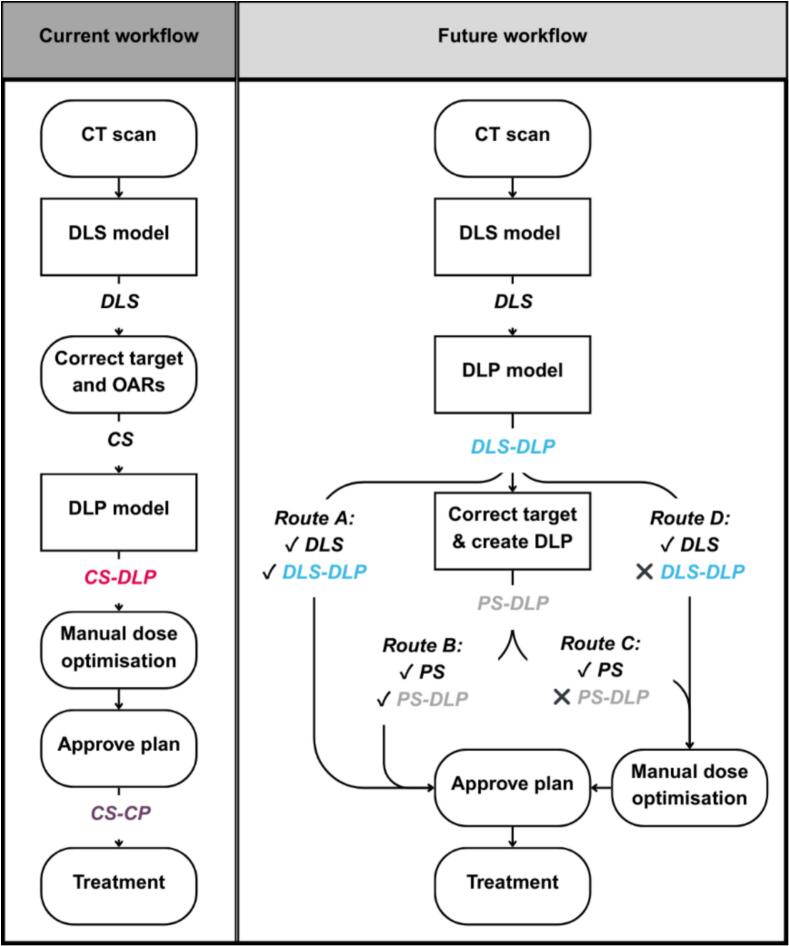
Schematic of the current and future workflow for left-sided node-negative breast cancer patients. In the future workflow, the radiation oncologist would evaluate the quality of the DL segmentations and plans and adjust where deemed necessary, which resulted in four potential routes. For the current and future routes t_active_ and t_inter_ were estimated with Supplementary Table S2 at: current (85 min − 37 h), A (20 min − 16 h), B (25 min − 24 h), C (75 min − 36 h), D (70 min − 36 h).

The DL planning workflow for left-sided node-negative breast cancer with a fractionation schedule of 15 fractions of 2.67 Gy was evaluated. The first plan setup in the current workflow (Fig. 1) was CS-DLP which used CS as input for the DL planning model, which resulted in a deep learning plan (DLP). Before it got clinically approved by the radiation oncologist (RO) and medical physicist (MP), CS-DLP could be optimised. This resulted in a clinically approved plan based on clinically approved structures, referred to as CS-CP, the gold standard.

Besides CS-DLP and CS-CP a third and fourth plan setup were created. It was hypothesised that these plan setups would reduce the time actively spent on treatment planning (t_active_) and the intermediate time between steps during working hours (t_inter_), while remaining clinically acceptable and similar to CS-CP. The third created plan was DLS-DLP, which used the deep learning structures (DLS) as input for the DL planning model, not allowing any alterations to the DLS nor DLP. Lastly, PS-DLP was created which used a set of Proposed Structures (PS) where only the DLS target volume would have been adjusted and clearly wrong DLS OARs, for example, if the heart were in the wrong location. In the data used, no clearly wrong DLS OARs were present. PS-DLP was created to assess the clinical impact of adjusting only the DLS target volume before creating a new DLP. An example of the four plan setups is given in Supplementary Fig. S1.

In addition to the current workflow (t_active_ = 85 min, t_inter_ = 37 h), a new potential workflow was also visualised in Fig. 1 that contains four potential workflow routes. Route A (t_active_ = 20 min, t_inter_ = 16 h) depicts the route where DLS-DLP got clinically approved without alterations. Routes B (t_active_ = 25 min, t_inter_ = 24 h) and C (t_active_ = 75 min, t_inter_ = 36 h) depict the routes where ROs would need to alter only the DLS CTVp and clearly wrong OARs (the proposed structures PS), and create a new DLP based on these new segmentations. For route B this new plan would not require any optimisation. For route C, this new plan would require manual optimisation by refining the DLP. It would also be possible to start over and create a dose distribution from scratch. Lastly, route D (t_active_ = 70 min, t_inter_ = 36 h) is the route where no adaptations were required to the DLS, however the DLP required manual optimisation similarly to route C. The times t_active_ and t_inter_ were derived from Supplementary Table S2 which contains time estimates provided by a group of daily users.

### 2.3 Evaluation criteria

Plan evaluation criteria for the PTVp-Skin05 were taken in accordance with the Dutch national consensus criteria: 39.65 Gy (99 %) ≤ D_mean_ ≤ 40.45 Gy (101 %), D_98_ ≥ 38.05 Gy (95 %), D_2_ ≤ 42.85 Gy (107 %) [16]. As these criteria do not specify dose-limiting goals for the OARs, three additional clinical goals were adopted from previously published studies: D_mean_ in Lungs ≤ 6 Gy, D_mean_ in Heart ≤ 3 Gy, D_mean_ in Breast CL ≤ 1 Gy [12], [17], [18], [19]. The number of clinical goals met per patient served as an indicator of plan performance. The evaluation of all plans was performed on the clinical structures: the gold standard.

In addition, tumour control probabilities (TCP) and normal tissue complication probabilities (NTCP) were determined to provide more direct estimates of therapeutic success and risks [20], [21], [22], [23]. The TCP was calculated using the EUD-based model of Gay and Niemierko [24] described in Arjmand et al. [25] with parameters from [24], [26], [27], [28], described in detail in Supplementary Material A.

For NTCP evaluation, the models of Darby et al. [17] and Taylor et al. [29] were identified as the most relevant indicators. The model of Darby et al. provided an estimate of the absolute excess risk (AER) of acute coronary events for patients both with and without pre-existing cardiac risk factors. The model of Taylor et al. estimated the absolute excess mortality risk (AEMR) due to secondary lung cancer or cardiac disease. In total six risk estimates were evaluated per plan setup per patient: twice for all three endpoints, once with and once without risk factor. An explanation of the methodology for both models is available in Supplementary Material A.

Differences between plan setups in DVH parameters, TCP and NTCP were statistically tested. The difference was tested for normality with the Shapiro-Wilk test for every combination between plan setups. As there was no metric where every difference was assumed to be normally distributed, the Friedman test was used to test for any significant differences. If significant differences were present, the Wilcoxon signed rank test was used with Bonferroni correction for multiple statistical testing.

For each patient it was decided retrospectively which route they would have taken to evaluate potential time savings. This was done with a set of decision rules. Patients would have taken route A if all six clinical goals were met in DLS-DLP. If not all goals were met for DLS-DLP, it was checked whether PS-DLP did. This simulated route B. If DLS-DLP and PS-DLP both did not reach all goals, it was assumed that the patient would have taken route C. For these time estimations no distinction was made between route C and D, as it would require manual evaluation of clinically relevant adjustments to the DLS. Instead, a worst-case scenario was assumed by taking route C as this route would require extra time to correct the DLS. TCP and NTCP were not taken into account for route decision making, as they were not used for clinical decision making in the current workflow.

## 3 Results

The gold standard, CS-CP, was the plan setup that achieved most clinical goals, 88 % of patients met all goals and 99 % met at least five (Table 2). Next, CS-DLP and PS-DLP performed clinically similar to each other as 59 % and 60 % of the patients met all clinical goals and 90 % and 87 % met at least five, respectively. The lowest number of patients meeting all goals was achieved by DLS-DLP: 43 %. However, 91 % of the patients still met 5 out of 6 goals. Additionally, there were two patients where DLS-DLP achieved more goals than CS-CP.

**Table 2:** DVH parameters for the six clinical goals for the four different plan setups. Goals met shows the percentage of the patients that met a certain number of the six clinical goals. Of the 101 patients the percentage is given for at least 6/6, 5/6, 4/6 and 3/6 goals met. None of the patients met less than three of the clinical goals. Additionally, the median (25th percentile (Q1) − 75th percentile (Q3)) were given. Breast CL: contralateral breast.

Evaluation metric		CS-CP	DLS-DLP	CS-DLP	PS-DLP
Minimum number of goals met [% of patients]	6/6 goals	88	43	59	60
	5/6 goals	99	90	90	87
	4/6 goals	100	96	99	99
	3/6 goals	100	100	100	100
PTVp-Skin05 − D_mean_ [Gy]		40.3 (40.2–––40.4)	40.4 (40.4–––40.4)	40.4 (40.4–––40.4)	40.4 (40.4–––40.4)
PTVp-Skin05 – D_98_ [Gy]		38.1 (38.1–––38.3)	38.2 (37.9–––38.4)	38.3 (38.1–––38.4)	38.3 (38.1–––38.4)
PTVp-Skin05 – D_2_ [Gy]		42.1 (41.9–––42.4)	42.2 (42.0–––42.4)	42.2 (42.0–––42.4)	42.2 (42.0–––42.4)
Lungs − D_mean_ [Gy]		2.3 (1.9–––2.7)	2.5 (2.1–––2.9)	2.3 (1.9–––2.7)	2.3 (1.9, 2.7)
Heart − D_mean_ [Gy]		1.1 (0.8–––1.4)	1.2 (0.9–––1.7)	1.1 (0.9–––1.6)	1.1 (0.8–––1.6)
Breast CL − D_mean_ [Gy]		0.3 (0.2–––0.4)	0.4 (0.3–––0.5)	0.3 (0.2––0.5)	0.3 (0.2–––0.5)

**Fig. 2:**
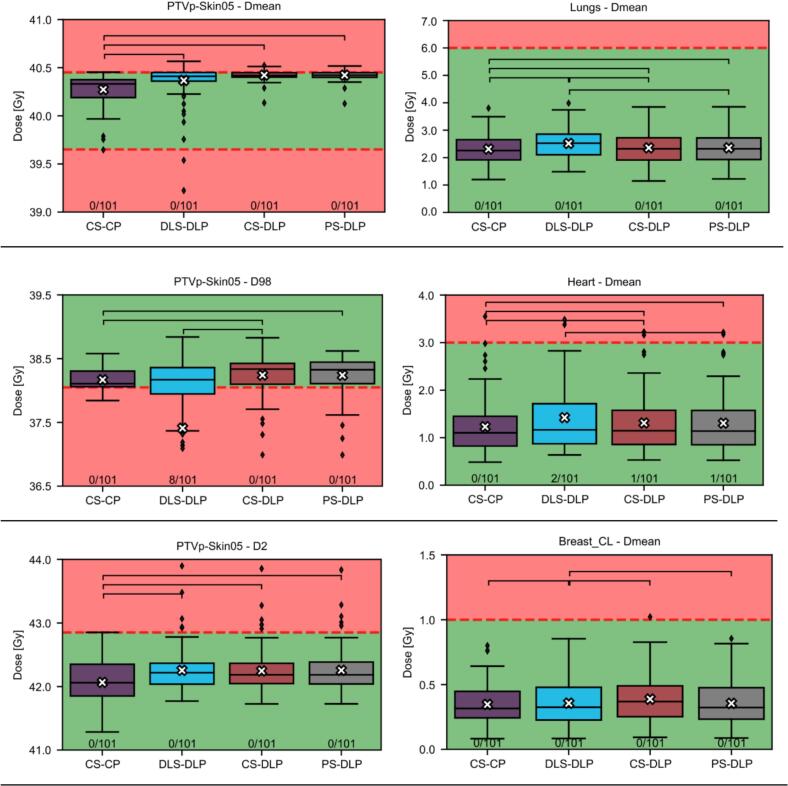
Boxplots for the clinical goals per plan setup. Green background: clinical goal met. Red background: clinical goal not met. The cross in the boxplot represents the mean. The fractions at the bottom represent how many patients are not visible within the current bounds of the plot. Braces represent statistically significant differences. (For interpretation of the references to colour in this figure legend, the reader is referred to the web version of this article.)

The DVH parameters were visualised in boxplots in Fig. 2 which showed the mean, out-of-bounds datapoints and statistically significant differences. Almost all DVH parameters showed statistically significant differences between plan setups (Fig. 2). However, only the D_98_ of PTV-Skin05 for DLS-DLP showed clinically relevant differences as it had a lower mean than the other three plan setups (CS-CP: 38.2 Gy, DLS-DLP: 37.4 Gy, CS-DLP: 38.3 Gy, PS-DLP: 38.3 Gy).

**Fig. 3:**
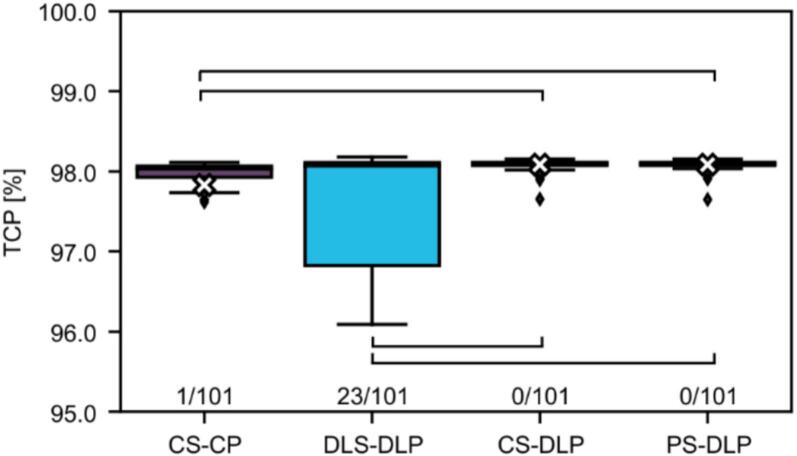
Boxplots showing the tumour control probability (TCP) for the four different plan setups. The cross in the boxplots represents the mean TCP. For DLS-DLP the mean is outside the bounds at 82 %. The fraction at the bottom of the figure shows the number of patients that have a TCP outside the bounds of the figure, all having a TCP lower than 95 %. Braces represent statistically significant differences.

The TCP of CS-DLP and PS-DLP were found to be highest and not statistically significantly different from each other (Fig. 3). Thereafter, the median TCP of DLS-DLP was higher than the TCP of CS-CP. This indicated that DLS-DLP produced a higher TCP for at least 50 % of the patients than the clinically used treatment plan. However, 23 % of the DLS-DLP plans had a TCP lower than 95 % which varied a lot from the other three plans (CS-CP: 1 %, CS-DLP: 0 %, PS-DLP: 0 %), resulting in an average TCP of 82 %. This indicated that for a large group of patients the adjustments to the target volume delineation increased the TCP.

The AER and AEMR (Table 3) showed that there is little difference in NTCP (order of 0.1 %) between the plan setups. However, multiple statistically significant differences were found, as shown in Supplementary Figs. S2 and S3.

**Table 3:** Absolute excess risk (AER) [17] and absolute excess mortality risk (AEMR) [29] for three endpoints, with (W) or without (WO) risk factor for the four different plan setups. For each combination the median (25th percentile (Q1) − 75th percentile (Q3)) are given.

NTCP − Endpoint	Risk Factor (W/WO)	CS-CP	DLS-DLP	CS-DLP	PS-DLP
AER [%] – Acute coronary events	W	0.7 (0.5–––0.9)	0.7 (0.5–––1.1)	0.7 (0.5 −, 1.0)	0.7 (0.5–––1.0)
	WO	0.4 (0.3–––0.5)	0.4 (0.3–––0.6)	0.4 (0.3–––0.5)	0.4 (0.3–––0.5)
AEMR [%] – Cardiac disease	W	0.4 (0.3–––0.5)	0.4 (0.3–––0.6)	0.4 (0.3–––0.5)	0.4 (0.3–––0.5)
	WO	0.1 (0.1–––0.1)	0.1 (0.1–––0.1)	0.1 (0.1–––0.1)	0.1 (0.1–––0.1)
AEMR [%] – Secondary lung cancer	W	2.3 (2.0–––2.7)	2.6 (2.2–––3.0)	2.4 (2.0–––2.8)	2.4 (2.0–––2.8)
	WO	0.1 (0.1–––0.1)	0.1 (0.1–––0.2)	0.1 (0.1–––0.1)	0.1 (0.1–––0.1)

Taking into account the set of decision rules, it was found that retrospectively of the patients, 43 % would have taken route A, 26 % route B and 32 % route C/D. This would have resulted in an average reduction per patient of 45 min for t_active_ and 12 h for t_inter_. The median, first quartile (Q1), and third quartile (Q3) values for the DVH parameters, TCP, and NTCP were reported for all four plan setups in Supplementary Tables S3A–C. Each table includes the subset of patients assigned to route A, B, or C/D, respectively.

## 4 Discussion

This research showed that for a large group of patients (43 %) no manual adjustments to DL segmentations and plans were needed to achieve a clinically acceptable plan with the clinical goals as evaluation criteria. Adjusting the segmentation of the target only and creating a new DLP would have sufficed for an additional 26 %. Only 32 % of all the patients required manual dose optimisation.

Considering radiobiological evaluation criteria it could be argued that DL-driven treatment plans were outperforming CS-CP. Starting with DLS-DLP having a median higher TCP than CS-CP despite having a clinically similar NTCP. However, the 23 out-of-bounds patients indicated that a large group of patients would have received a too low TCP. The inclusion of PS-DLP showed that for these 23 patients adjusting the CTVp DLS and creating a new DLP would lead to clinically acceptable TCP and NTCP values, similar and sometimes even better than CS-CP.

If one would completely ignore the clinical goals, the inclusion of TCP and NTCP highlighted three things. Firstly, that manual optimisations to the created DL plan had little clinical advantage. Secondly, manual adjustments to the DL segmentations of the OARs were clinically irrelevant in all cases. Lastly, adjustments to the DL segmentation of only the CTVp were clinically relevant for 23 % of cases.

It could be seen that manual adjustments to a DL plan increased the number of clinical goals met, as 88 % of CS-CP plans met all clinical goals compared to 59 % for CS-DLP. It was known that experts put effort into reaching the clinical goals, even when the current goal was only off by a few cGy. However, as Fig. 3 and Supplementary Figs. S2 and S3 show, this small difference in cGy did not translate to a clinically relevant improvement in TCP and NTCP. In an earlier stage of this research more NTCP endpoints were investigated (e.g., heart pericarditis, secondary cancer risk for heart and lung pneumonitis). However, as these NTCP values were negligible (<0.1 %) they were not included in later stages of the research.

There were two patients where DLS-DLP met more clinical goals than CS-CP. This suggests that manual adjustments to the DL outputs reduced plan quality. However, this interpretation would be too narrow as ROs assess clinical acceptability using not only predefined clinical goals, but also patient‑specific considerations not present in a dose distribution (e.g., age, medical history). As a result, a plan may fail to meet all clinical goals yet still be deemed clinically acceptable.

Besouw et al. [12] reported that utilising mimic settings generalised over institutes would result in 77 % of CS-DLP plans meeting all clinical goals. Similarly, Bakx et al. [9] reported that 80 % of test patients met all clinical goals when creating CS-DLP plans. These results reported better results in terms of clinical goals met. This was caused by the usage of different DL planning model versions. This new version (RayStation 2024B) of the DL planning model was known to create plans with a higher D_mean_ and D_2_, which caused more plans not to reach the associated clinical goals.

Separate versions of RayStation, with varying DL models, were used when creating CS-CP. Both the segmentations and plans needed to be approved by experts before being clinically accepted. During the time period of this patient cohort, no changes were made to the evaluation criteria. Therefore, it can be ensured that CS-CP could be used as gold standard, even though there might be DL-induced variations dependent on model version [30].

The TCP model used parameters that corresponded to a 2 Gy per fraction fractionation schedule. Therefore, there might be a deviation from the true TCP. However, Arjmand et al. [25] used the same parameters to determine the TCP for the fractionation schedule used for this patient cohort showing that it is still applicable. Especially as they note that this was accounted for by using an equivalent uniform dose concept. Besides, the same model was used for all four plan setups, and the possible inaccuracy would affect all plan setups equally. Moreover, TCP models assume homogeneous tumour cell distribution within the breast and therefore TCP values should be evaluated with care. This assumption causes a larger uncertainty in the TCP than a small deviation in fractionation schedule.

One of the limitations of this research was that no qualitative analysis was incorporated, as encouraged by Vandewinckele et al. [6] and Bakx et al. [9]. Both mention how a qualitative analysis helps to evaluate the clinical acceptability of DL plans by expert scoring. However, one may question how clinically relevant these observations are if a proper set of DVH criteria together with TCP and NTCP criteria would already be used for plan evaluation.

The contralateral breast was not part of the organs-at-risk for earlier treated patients and therefore was only available as CS 12 times. However, the contralateral breasts needed little corrections as Table 1 and previous studies in the institute have shown [14]. Therefore, the DL segmentation was used if the patient did not have a CS contralateral breast. This assumption did not lead to different results.

It can be assumed that the ROs will need some time to become accustomed to the new workflow: they will be provided with a plan, need to evaluate the DLS quality and whether adjustments would improve the plan. Therefore, for future target DLS that are adapted, a DLS-DLP will also be created to see how much the adaptation influenced the plan and return this information to the RO as a continuous information feedback loop. This will help the RO to see when corrections were useful. Additionally, correctly evaluating a DLS-DLP plan on clinical acceptability would greatly benefit an online adaptive radiotherapy approach [31].

To assist the user, a tool could be designed that would alert the user that this patient might benefit from manual adjustments to the target volume based on segmentation prediction uncertainty maps as proposed by e.g., Huet-Dastarac et al. [32]. Next to uncertainty maps it was also hypothesised that a correlation between geometric metrics and DVH parameters could help indicate clinically relevant adaptations. Unfortunately, we were unable to detect a correlation.

In conclusion, a potential workflow for radiotherapy treatment planning for left-sided node-negative breast cancer was retrospectively evaluated. It was found that this new workflow would on average reduce the active time per patient by 45 min and intermediate time by 12 h based on estimated time saving within the institute. Additionally, the methodology could be applied to other tumour sites with DL segmentation and planning models.

## CRediT authorship contribution statement

**Niels van Acht:** Writing – review & editing, Writing – original draft, Visualization, Validation, Software, Methodology, Formal analysis, Conceptualization. **Andreea Ciobotaru:** Writing – review & editing, Methodology, Investigation, Formal analysis. **Maurice van der Sangen:** Writing – review & editing, Validation, Conceptualization. **Jacqueline Theuws:** Writing – review & editing, Validation. **Johanna Bluemink:** Writing – review & editing. **Coen Hurkmans:** Writing – review & editing, Methodology, Conceptualization.

## Declaration of competing interest

The authors declare the following financial interests/personal relationships which may be considered as potential competing interests: A research grant from RaySearch Laboratories AB for Niels van Acht is gratefully acknowledged. Coen Hurkmans is an Editorial Board Member for this journal and was not involved in the editorial review or decision to publish this article.
