# Supplementary material for: Automated deep learning segmentation and planning for left-sided breast radiotherapy with minimised adaptations based on dose, TCP and NTCP criteria
Source: Phys Imaging Radiat Oncol. 2026 Apr 4;38:100961. doi: 10.1016/j.phro.2026.100961 (PMC13091286; doi:10.1016/j.phro.2026.100961)
Supplement: Supplementary Data 1 [file mmc1.pdf]

Supplementary material A

Figures

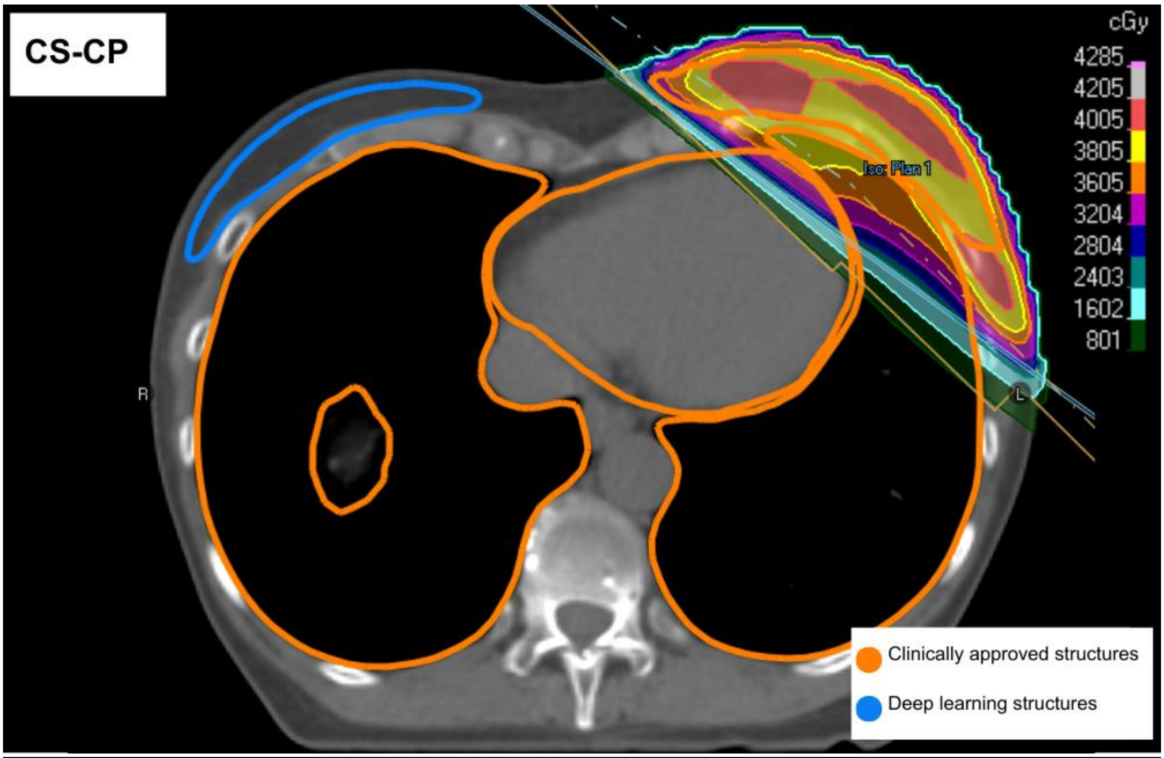

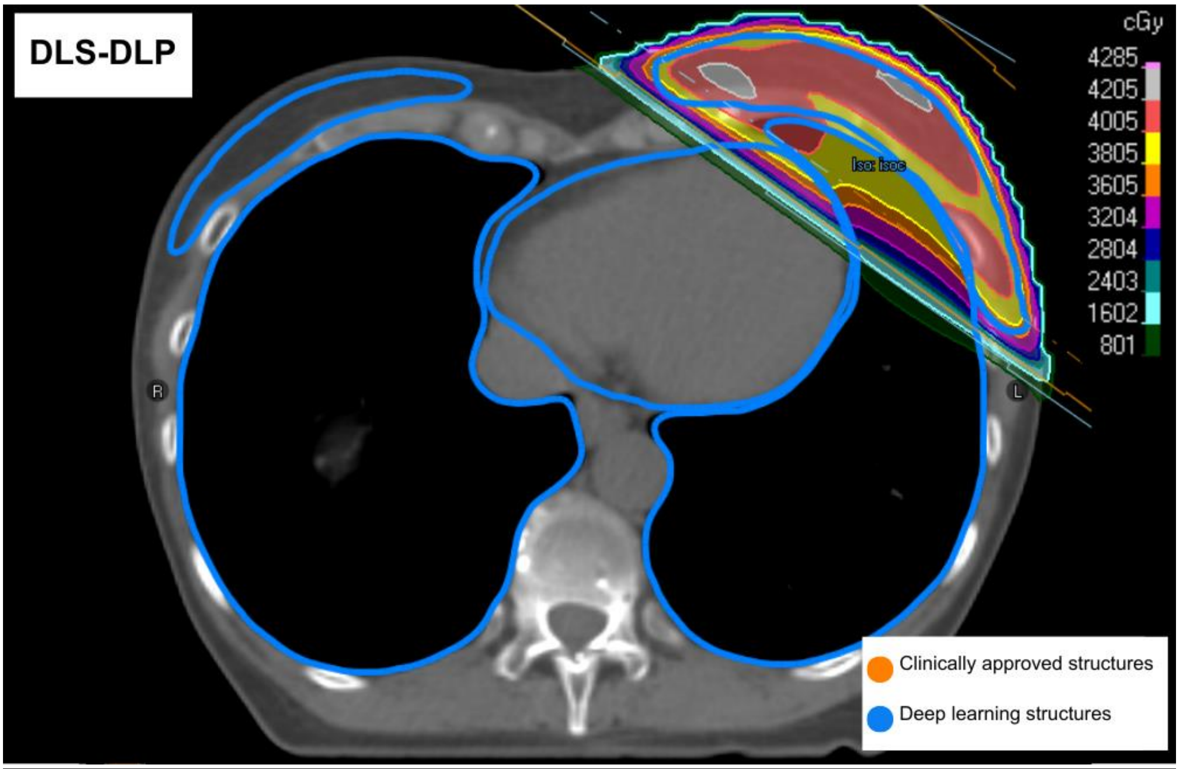

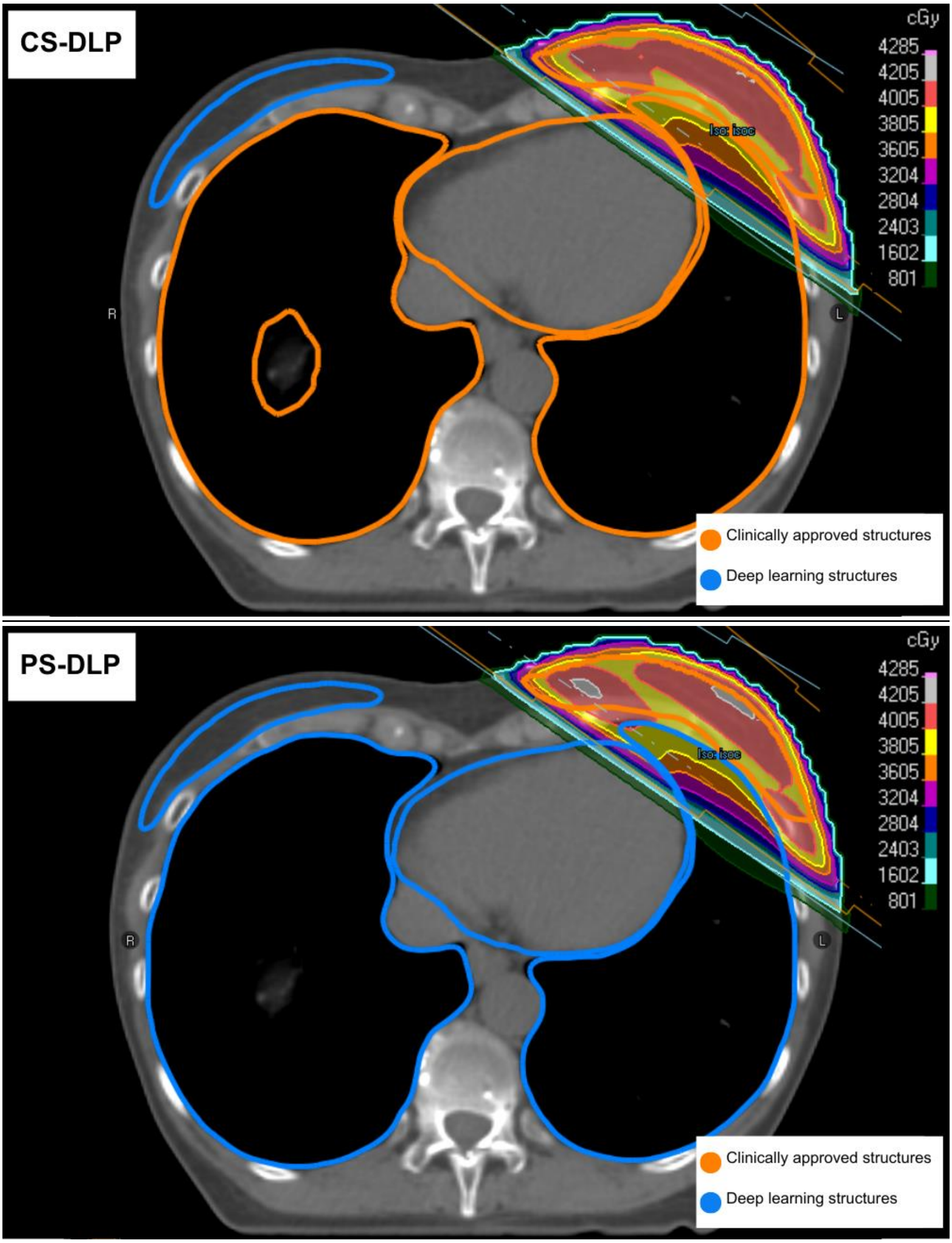

Supplementary Figure S1: Screenshot of four dose distributions of an anonymised patient. The orange structures indicate clinically approved structures (CS). Blue structures indicate deep learning structures (DLS). The structures visible in each subfigure were the structures used to create that dose distribution, where there is a special case that uses the proposed structures (PS). PS consisted of the clinically approved structure for the CTVp and the deep learning structures for the organs-at-risk. All screenshots were taken on the same slice. For this patient no CS of the contralateral breast was present, therefore the DLS was taken as substitute.

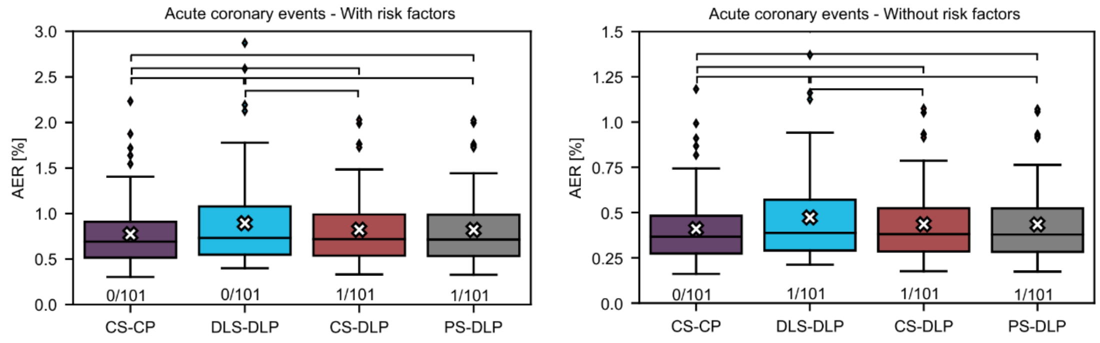

Supplementary Figure S2: Boxplots for the absolute excess risk of acute coronary events. Separate figures are given with (left column) or without (right column) baseline risk factors. The cross in the boxplot represents the mean. The fractions at the bottom represents how many patients are not visible within the current bounds of the plot. Braces represent statistically significant differences.

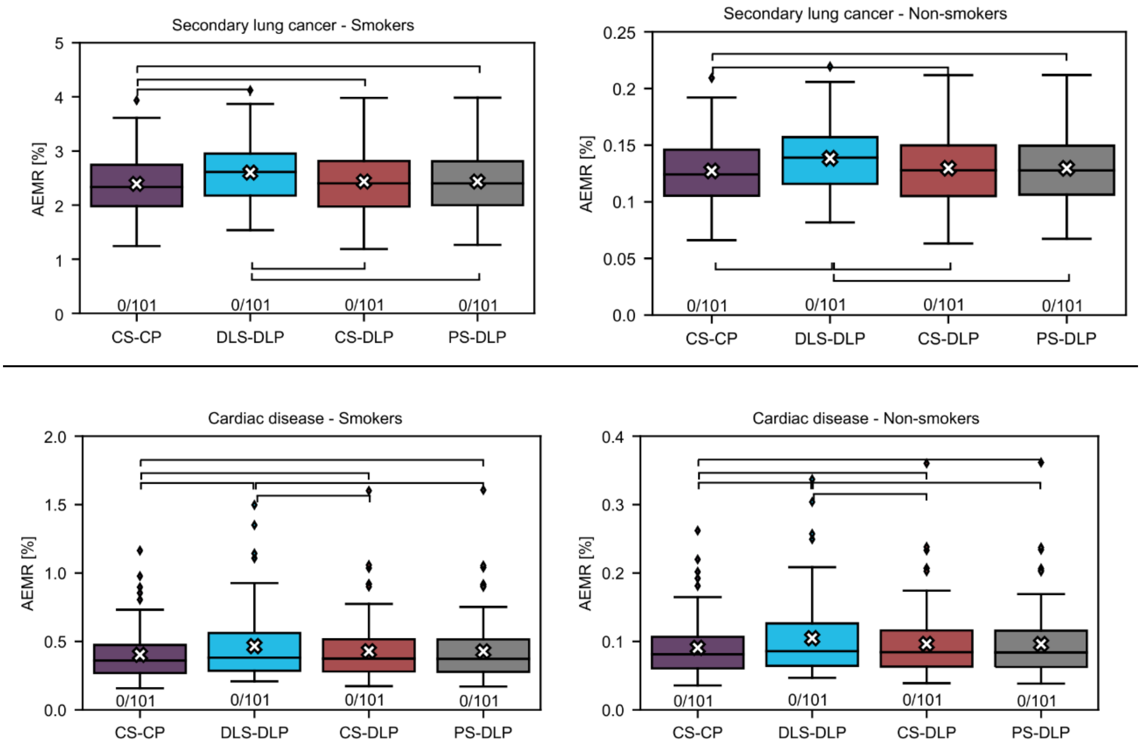

Supplementary Figure S3: Boxplots for the absolute excess mortality risk due to secondary lung cancer (top row) or due to cardiac disease (bottom row). Separate figures are given for smokers and non-smokers as baseline risk factor. The cross in the boxplot represents the mean. The fractions at the bottom represents how many patients are not visible within the current bounds of the plot. Braces represent statistically significant differences.

Tables

Supplementary Table S1: nomenclature table with the abbreviations used in the article.

Abbreviation	Definition
VDSC	Volumetric Dice similarity coefficient
SDSC	Surface Dice similarity coefficient with 3 mm tolerance
HD95	95th percentile Hausdorff distance
APL	Added path length
D_{mean}	Average dose in a ROI
D_{98}	Dose received in 98 % of the volume of a ROI
D_{2}	Dose received in 2 % of the volume of a ROI
TCP	Tumour control probability
NTCP	Normal tissue complication probability
AER	Absolute excess risk
AEMR	Absolute excess mortality risk
CS	Clinically approved structures
DLS	Deep learning structures
PS	Proposed structures: Consists of the clinically approved structure for the CTVp and the deep learning structures for the organs-at-risk
CP	Clinically approved plan
DLP	Deep learning plan
CS-CP	Clinically approved plan created using clinically approved structures
CS-DLP	Deep learning plan created using clinically approved structures
DLS-DLP	Deep learning plan created using deep learning structures
PS-DLP	Deep learning plan created using proposed structures.

Supplementary Table S2: Actions used in the workflow to create a treatment plan. Per step in the workflow estimates are given for the active and intermediate time. These time estimates were given by a group of clinicians that work with this workflow on a daily basis. The estimates were used to calculate the time spent on the routes mentioned in Figure 1. When a range was reported instead of a fixed time the average was used in the calculation. Intermediate time was given in hours where a full day of work is 8 hours.

Actions in workflow	Active time	Intermediate time
RTT: create DLS	1 minute	0.5-1 hours
RTT: adjust OARs	5-10 minutes	-
RO: adjust CTVp	10-20 minutes	8-24 hours
RTT: create DLP	5 minutes	1-8 hours
RTT: adjust DLP	10-60 minutes	-
RTT: check plan by different RTT	10 minutes	1-8 hours
RO: check and approve plan	10 minutes	4-12 hours
MP: check and approve plan	5 minutes	1-8 hours

Supplementary Table S3A: Summary of evaluation criteria for all patients that would have followed route A (all clinical goals met for DLS-DLP). The median (25th percentile (Q1) - 75th percentile (Q3)) are given for the DVH parameters, TCP and NTCPs.

**Route A patients (N=43)**		**CS-CP**	**DLS-DLP**	**CS-DLP**	**PS-DLP**
**DVH parameters**					
PTVp-Skin05 - D_{mean} [Gy]		40.4 (40.3 - 40.4)	40.4 (40.4 - 40.4)	40.4 (40.4 - 40.4)	40.4 (40.4 - 40.4)
PTVp-Skin05 – D_{98} [Gy]		38.1 (38.1 - 38.3)	38.3 (38.2 - 38.4)	38.4 (38.2 - 38.4)	38.3 (38.2 - 38.4)
PTVp-Skin05 – D_2 [Gy]		42.0 (41.9 - 42.2)	42.1 (42.0 - 42.3)	42.1 (42.0 - 42.3)	42.2 (42.0 - 42.3)
Lungs - D_{mean} [Gy]		2.4 (1.9 - 2.6)	2.5 (2.1 - 2.9)	2.4 (2.0 - 2.7)	2.4 (2.0 - 2.7)
Heart - D_{mean} [Gy]		1.0 (0.8 - 1.5)	1.2 (1.0 - 1.7)	1.1 (0.9 - 1.5)	1.1 (0.9 - 1.5)
Breast CL - D_{mean} [Gy]		0.3 (0.3 - 0.5)	0.4 (0.3 - 0.5)	0.3 (0.2 - 0.5)	0.3 (0.2 - 0.5)
**TCP**		98.1 (98.0 - 98.1)	98.1 (98.0 - 98.1)	98.1 (98.1 - 98.1)	98.1 (98.1 - 98.1)
**NTCP**	**Risk Factor (W/WO)**				
AER [%] – Acute coronary events	W	0.7 (0.5 - 0.9)	0.7 (0.6 - 1.0)	0.7 (0.6 - 0.9)	0.7 (0.6 - 0.9)
	WO	0.3 (0.3 - 0.5)	0.4 (0.3 - 0.6)	0.4 (0.3 - 0.5)	0.4 (0.3 - 0.5)
AEMR [%] – Cardiac disease	W	0.3 (0.3 - 0.5)	0.4 (0.3 - 0.5)	0.4 (0.3 - 0.5)	0.4 (0.3 - 0.5)
	WO	0.1 (0.1 - 0.1)	0.1 (0.1 - 0.1)	0.1 (0.1 - 0.1)	0.1 (0.1 - 0.1)
AEMR [%] – Secondary lung cancer	W	2.5 (2.0 - 2.7)	2.6 (2.2 - 3.0)	2.5 (2.0 - 2.8)	2.5 (2.1 - 2.8)
	WO	0.1 (0.1 - 0.1)	0.1 (0.1 - 0.2)	0.1 (0.1 - 0.1)	0.1 (0.1 - 0.1)

Supplementary Table S3B: Summary of evaluation criteria for all patients that would have followed route B (All clinical goals met for PS-DLP, when DLS-DLP did not). The median (25th percentile (Q1) - 75th percentile (Q3)) are given for the DVH parameters, TCP and NTCPs.

Route B patients (N=26)		CS-CP	DLS-DLP	CS-DLP	PS-DLP
**DVH parameters**					
PTVp-Skin05 - D_{mean} [Gy]		40.3 (40.1 - 40.3)	40.4 (40.3 - 40.5)	40.4 (40.4 - 40.4)	40.4 (40.4 - 40.4)
PTVp-Skin05 – D_{98} [Gy]		38.1 (38.1 - 38.2)	38.0 (37.5 - 38.3)	38.4 (38.2 - 38.4)	38.4 (38.2 - 38.5)
PTVp-Skin05 – D_{2} [Gy]		42.0 (41.7 - 42.2)	42.2 (42.1 - 42.3)	42.2 (42.1 - 42.3)	42.1 (42.0 - 42.2)
Lungs - D_{mean} [Gy]		2.2 (1.8 - 2.4)	2.3 (2.0 - 2.8)	2.3 (1.8 - 2.6)	2.3 (1.9 - 2.6)
Heart - D_{mean} [Gy]		0.9 (0.8 - 1.4)	1.0 (0.8 - 1.4)	1.0 (0.8 - 1.5)	1.0 (0.8 - 1.5)
Breast CL - D_{mean} [Gy]		0.3 (0.3 - 0.4)	0.4 (0.3 - 0.5)	0.4 (0.3 - 0.5)	0.4 (0.3 - 0.5)
**TCP**		98.0 (97.9 - 98.0)	97.9 (69.3 - 98.1)	98.1 (98.1 - 98.1)	98.1 (98.1 - 98.1)
**NTCP**	**Risk Factor (W/WO)**				
AER [%] – Acute coronary events	W	0.6 (0.5 - 0.9)	0.6 (0.5 - 0.9)	0.6 (0.5 - 0.9)	0.6 (0.5 - 0.9)
	WO	0.3 (0.3 - 0.5)	0.3 (0.3 - 0.5)	0.3 (0.3 - 0.5)	0.3 (0.3 - 0.5)
AEMR [%] – Cardiac disease	W	0.3 (0.3 - 0.4)	0.3 (0.3 - 0.5)	0.3 (0.3 - 0.5)	0.3 (0.3 - 0.5)
	WO	0.1 (0.1 - 0.1)	0.1 (0.1 - 0.1)	0.1 (0.1 - 0.1)	0.1 (0.1 - 0.1)
AEMR [%] – Secondary lung cancer	W	2.3 (1.8 - 2.5)	2.4 (2.1 - 2.9)	2.4 (1.9 - 2.6)	2.4 (1.9 - 2.6)
	WO	0.1 (0.1 - 0.1)	0.1 (0.1 - 0.2)	0.1 (0.1 - 0.1)	0.1 (0.1 - 0.1)

Supplementary Table S3C: Summary of evaluation criteria for all patients that would have followed route C or D (Both the DLS-DLP and PS-DLP plans did not meet all clinical goals). The median (25th percentile (Q1) - 75th percentile (Q3)) are given for the DVH parameters, TCP and NTCPs.

Route C/D patients (N=32)		CS-CP	DLS-DLP	CS-DLP	PS-DLP
**DVH parameters**					
PTVp-Skin05 - D_{mean} [Gy]		40.3 (40.2 - 40.4)	40.4 (40.3 - 40.5)	40.5 (40.4 - 40.5)	40.5 (40.4 - 40.5)
PTVp-Skin05 – D_{98} [Gy]		38.1 (38.1 - 38.1)	38.0 (37.5 - 38.3)	38.1 (37.9 - 38.4)	38.0 (37.8 - 38.4)
PTVp-Skin05 – D_{2} [Gy]		42.2 (41.9 - 42.4)	42.3 (42.1 - 42.7)	42.3 (42.1 - 42.7)	42.3 (42.1 - 42.7)
Lungs - D_{mean} [Gy]		2.2 (2.0 - 2.9)	2.6 (2.2 - 2.9)	2.3 (2.0 - 2.9)	2.3 (2.0 - 2.9)
Heart - D_{mean} [Gy]		1.1 (0.8 - 1.6)	1.3 (0.9 - 2.2)	1.2 (0.8 - 1.6)	1.2 (0.8 - 1.6)
Breast CL - D_{mean} [Gy]		0.3 (0.2 - 0.3)	0.3 (0.2 - 0.4)	0.3 (0.2 - 0.4)	0.3 (0.2 - 0.4)
**TCP**		98.0 (97.9 - 98.1)	98.1 (27.7 - 98.1)	98.1 (98.0 - 98.1)	98.1 (98.0 - 98.1)
**NTCP**	**Risk Factor (W/WO)**				
AER [%] – Acute coronary events	W	0.7 (0.5 - 1.0)	0.8 (0.6 - 1.4)	0.8 (0.5 - 1.0)	0.8 (0.5 - 1.0)
	WO	0.4 (0.3 - 0.5)	0.4 (0.3 - 0.7)	0.4 (0.3 - 0.5)	0.4 (0.3 - 0.5)
AEMR [%] – Cardiac disease	W	0.4 (0.3 - 0.5)	0.4 (0.3 - 0.7)	0.4 (0.3 - 0.5)	0.4 (0.3 - 0.5)
	WO	0.1 (0.1 - 0.1)	0.1 (0.1 - 0.2)	0.1 (0.1 - 0.1)	0.1 (0.1 - 0.1)
AEMR [%] – Secondary lung cancer	W	2.3 (2.0 - 3.0)	2.7 (2.3 - 3.0)	2.4 (2.0 - 3.0)	2.4 (2.0 - 3.0)
	WO	0.1 (0.1 - 0.2)	0.1 (0.1 - 0.2)	0.1 (0.1 - 0.2)	0.1 (0.1 - 0.2)

Tumour control probability and normal tissue complication probability models

Tumour control probability (TCP) is an important radiobiological metric that describes the probability that local tumour control is achieved. Gay and Niemierko [24] proposed a method to calculate the TCP using the equivalent uniform dose (EUD) of a treatment plan which was calculated as

$$EUD = \left(\sum_{i=1}\left(v_i \cdot EQD_{2,i}{}^{a}\right)\right)^{1/a}, \tag{1}$$

where

$$EQD_{2,i} = D_i \frac{\left(\alpha/\beta\right) + d_i}{\left(\alpha/\beta\right) + 2}. \tag{2}$$

In equation (1), v_i describes the unitless i'th partial volume receiving dose D_i in Gy. The model parameter a is specific to the tumour of interest. For this research, a was -7.2 as provided by Gay and Niemierko [24]. In equation (2), α/β describes a tissue's sensitivity to radiation dose fractionation which was set at 3 Gy [27, 28]. d_i is the dose received by v_i per fraction. Next, the EUD was used in the model to determine the TCP as

$$TCP = \left(1 + \left(\frac{TCD_{50}}{EUD}\right)^{4\gamma_{50}}\right)^{-1}. \tag{3}$$

In equation (3), TCD_{50} is the tumour dose required to control 50 % of the tumours when the tumour is homogeneously irradiated. γ_{50} is another model parameter, specific for the tumour of interest. For this research TCD_{50} and γ_{50} were 28 Gy [26] and 2 [24] respectively.

Next, data from Darby et al. [17] and Taylor et al. [29] were used to determine the normal tissue complication probability (NTCP). Darby et al. [17] described a method to estimate the absolute excess risk (AER) for any acute coronary events (ACE) with

$$AER = BR \cdot ERR \cdot D_{mean}. \tag{4}$$

Equation (4) uses the baseline risk (BR), excess relative risk (ERR) and the mean dose (D_{mean}) in the region-of-interest of the endpoint. They described that BR varied per endpoint and was dependent on whether the patient had any risk factors. The BR was 8.5 % and 4.5 % for patients with and without risk factors respectively. The ERR was determined to be 0.074 per Gy of mean heart dose.

Taylor et al. [29] described a method to estimate the absolute excess mortality risk (AEMR) for the endpoints of mortality due to secondary lung cancer and cardiac mortality after breast cancer radiotherapy. Equation (4) was used by them as well to determine the AEMR instead of AER. For mortality due to secondary lung cancer the BR was 9.4 % and 0.5 % for patients with and without risk factors respectively. For cardiac mortality these values were 8.0 % and 1.8 %. Additionally, the ERR was determined to be 0.11 per Gy of mean lung dose and 0.041 per Gy of mean heart dose.

Both the AER and AEMR estimates were evaluated for patients at an age of 80, with an attained age of 50.

Deep learning segmentation and planning model summary

This research utilised the commercially available deep learning segmentation and deep learning planning model in RayStation, the treatment planning system of RaySearch Laboratories AB. The patient’s treatment plans were created in v9B (01-10-2020 to 05-04-2022), v10B (05-04-2022 to 11-05-2023), v12A (11-05-2023 to 12-12-2024) and v2024B (12-12-2024 and onwards).

The deep learning segmentation model was a 3D U-Net CNN that had as input a CT scan of the patient. It is within the intended use that the patients were imaged in the treatment position, which was head-first-supine with arms up for this research. It is not within the intended use to utilise image artefact reduction algorithms. Therefore, no patients were included in the dataset where this was the case. Lastly, no patients that had undergone a mastectomy were present in the training dataset. Contours of the regions-of-interests were received as output of the deep learning segmentation model. For this research the regions-of-interests were:

- Breast_L
- Breast_R
- Lung_L
- Lung_R
- Heart

After receiving the deep learning structures, Breast_L was renamed to CTVp and Breast_R to Breast_CL. Lastly, Lung_L and Lung_R were combined and renamed to Lungs.

The deep learning model was a 3D U-Net CNN with 5 input channels that require binary contours of:

- PTV: The CTVp extended by 10 mm in all directions.
- PTV-Skin: PTV excluding the external. For this research PTVp-Skin05 was used here, which is the PTV at least 5 mm away from the external.
- Heart
- Lungs
- External

The primary description of the training data was 40.05 Gy, delivered in 15 fractions of 2.67 Gy. The treatment technique was SMLC. The patients in the training contained beams of either 6 or 10 MV. The model was trained on patients with the protocol described in Supplementary Table S4.

The deep learning planning model gives a dose prediction as output with a prescribed dose per voxel. This dose prediction was followed by dose mimicking optimisation, aiming to achieve a deliverable treatment plan with a dose distribution similar to the predicted dose.

Supplementary Table S4: Clinical goals corresponding to the protocol used for training the deep learning planning model.

ROI	Clinical goal
PTV-Skin	At least 38.05 Gy dose at 98 % volume
PTV-Skin	At least 40.05 Gy average dose
PTV-Skin	At most 4.85 Gy dose at 2 % volume
Heart	At most 16 Gy dose at 5 % volume
Heart	At most 8 Gy dose at 30 % volume
Heart	At most 3.2 Gy average dose
Lung_L	At most 16 Gy dose at 15 % volume
Lung_L	At most 8 Gy dose at 35 % volume
Lung_L	At most 4 Gy dose at 50 % volume
Lung_R	At most 4 Gy dose at 10 % volume
